# Bis-Quinolinium Cyclophane Blockers of SK Potassium Channels Are Antagonists of M3 Muscarinic Acetylcholine Receptors

**DOI:** 10.3389/fphar.2020.552211

**Published:** 2020-09-16

**Authors:** Vladislav Bugay, Derek J. Wallace, Bin Wang, Irving Salinas, Adriana Paola Chapparo, Hudson Ryan Smith, Peter Herbert Dube, Edward G. Brooks, Kelly Ann Berg, Robert Brenner

**Affiliations:** ^1^ Cell and Integrative Physiology, UT Health San Antonio, San Antonio, TX, United States; ^2^ Intensive Care Unit, Methodist Hospital Texsan, San Antonio, TX, United States; ^3^ Department of Physiology, Michigan State University, East Lansing, MI, United States; ^4^ Department of Pediatrics, UT Health San Antonio, San Antonio, TX, United States; ^5^ Department of Pharmacology, UT Health San Antonio, San Antonio, TX, United States; ^6^ Microbiology, Immunology & Molecular Genetics, UT Health San Antonio, San Antonio, TX, United States

**Keywords:** muscarinic receptor, SK channel, dequalinium, UCL 1684, airway smooth muscle, contraction

## Abstract

Dequalinium is used as an antimicrobial compound for oral health and other microbial infections. Derivatives of dequalinium, the bis-quinolinium cyclophanes UCL 1684 and UCL 1848, are high affinity SK potassium channel antagonists. Here we investigated these compounds as M3 muscarinic receptor (mACHR) antagonists. We used the R-CEPIAer endoplasmic reticulum calcium reporter to functionally assay for Gq-coupled receptor signaling, and investigated the bis-quinolinium cyclophanes as antagonists of M3 mACHR activation in transfected CHO cells. Given mACHR roles in airway smooth muscle (ASM) contractility, we also tested the ability of UCL 1684 to relax ASM. We find that these compounds antagonized M3 mACHRs with an IC_50_ of 0.27 μM for dequalinium chloride, 1.5 μM for UCL 1684 and 1.0 μM for UCL 1848. UCL 1684 also antagonized M1 (IC_50_ 0.12 μM) and M5 (IC_50_ 0.52 μM) mACHR responses. UCL 1684 was determined to be a competitive antagonist at M3 receptors as it increased the EC_50_ for carbachol without a reduction in the maximum response. The Ki for UCL1684 determined from competition binding experiments was 909 nM. UCL 1684 reduced carbachol-evoked ASM contractions (>90%, IC_50_ 0.43 μM), and calcium mobilization in rodent and human lung ASM cells. We conclude that dequalinium and bis-quinolinium cyclophanes antagonized M3 mACHR activation at sub- to low micromolar concentrations, with UCL 1684 acting as an ASM relaxant. Caution should be taken when using these compounds to block SK potassium channels, as inhibition of mACHRs may be a side-effect if excessive concentrations are used.

## Introduction

Dequalinium salts were first described as antibacterial agents (Babbs et al., 1956), that are currently in use in oral disinfectant mouthwashes (i.e. Dequadin and others) (Kaufman, 1981; Griffiths and Stock, 1986; Liberman et al., 1989), and also for treatment of vaginal bacterial infections (Petersen et al., 2002; Mendling et al., 2016). More recently, dequalinium has been shown to accumulate in mitochondria (Modica-Napolitano and Aprille, 2001) which may underlie its antitumor activity (Weiss et al., 1987; Christman et al., 1990), but also has been useful as a mitochondrial targeting agent for chemotherapeutics (Weissig and Torchilin, 2001) and gene delivery (Bae et al., 2017).

Given the potential therapeutic uses of dequalinium compounds, it is important to understand their binding targets. Dequalinium modifies the activity of a number of receptors and ion channels. This was first evidenced by early studies showing that dequalinium had paralyzing activity after injection into mice and rabbits (Collier and Taylor, 1949). Consistent with its paralyzing activity, studies in frog and rat sympathetic neurons directly showed that dequalinium reduced cholinergic neurotransmission, and responses to nicotinic receptor agonists (Dunn, 1993). Others showed that while dequalinium could block nicotinic responses in skeletal muscle, it was also an effective blocker of SK potassium channels (IC_50_ 1.5 μM) (Castle et al., 1993; Dunn, 1994). Given that dequalinium was the first synthetic compound that could block SK potassium channels at micromolar concentrations, it was used as a template for modifications that would increase affinity for SK channels (Galanakis et al., 1995; Dunn et al., 1996; Galanakis et al., 1996). These efforts led to the synthesis of compounds that could block SK channels at nanomolar concentrations, and included the dequalinium-cyclophanes UCL 1684 (IC_50_ 3 nM) (Campos Rosa et al., 1996) and UCL 1848 (IC_50_ 2.7 nM) (Chen et al., 2000). UCL 1684, and to a lesser extent, UCL 1848, have been extensively employed as alternatives to the SK channel antagonist peptide, Apamin, to interrogate the physiological roles of SK channels and as potential therapeutic agents (Liegeois et al., 2003; Blank et al., 2004). Dequalinium was also shown to block cyclic nucleotide-gated channels with IC_50_ of 190 nM for CNG1 and 2.4 μM for CNG2 channels (Rosenbaum et al., 2004). More recently, it has been found that dequalinium and UCL 1684 also inhibit TrpM3 and TrpM7 channels (~30%–50% reduction) albeit at much lower affinity (30 μM concentration) (Chubanov et al., 2012).

Given the common use of UCL 1684 to block SK channels, and early studies suggesting that dequalinium may also antagonize muscarinic responses (Dunn, 1993), we investigated UCL 1684 interactions with muscarinic acetylcholine receptors. We focused on M3 muscarinic acetylcholine receptors since these receptors have broad roles in smooth muscle contractility in many organs including airway, bladder and gut smooth muscle. Our primary goal was to characterize UCL 1684 pharmacology with transfected muscarinic receptors in CHO cells, and corroborate these potential interactions using physiological studies in airway smooth muscle.

## Materials and Methods

### Tracheal Tension Measurements

Mice used in these studies were BALB/C mice from Jackson Labs. All animal procedures were reviewed and approved by the University of Texas Health Science Center at San Antonio Institutional Animal Care and Use Committee. For tracheal constriction studies, animals were deeply anesthetized with isoflurane and then sacrificed by cervical dislocation. Isometric tracheal contraction measurements were performed as previously described (Semenov et al., 2012). Trachea were removed and surrounding tissues dissected in ice-cold physiologic saline solution (PSS) consisting of 119 mM NaCl, 4.7 mM KCl, 2.0 mM CaCl_2_, 1.0 mM KH_2_PO_4_, 1.17 mM MgSO_4_, 18 mM NaHCO_3_, 0.026 mM EDTA, 11 mM glucose, and 12.5 mM sucrose. Trachea were cut below the pharynx and above the primary bronchus bifurcation. Two metal wires, attached to a force transducer and micrometer (Radnoti, LLC), were threaded into the lumen of the trachea. Trachea were placed into an oxygenated organ bath (95% O_2_, 5% CO_2_), at 37°C, pH 7.35 (after bubbling with 95% O_2_–5% CO_2_). Tracheal resting tension was readjusted to 1 gram over 1 h and then challenged with 67 mM potassium PSS with NaCl adjusted to 56.7 mM to maintain proper osmolarity (KPS). Trachea were challenged two or more times until reproducible contractile responses were achieved. All subsequent experimental drug responses were normalized to the KPS response. Bladder measurements were conducted in a similar manner as trachea with identical solutions. The bladder was excised and formed into a ring by cutting anterior and posterior portions before mounting on metal wires for force measurements. For the aorta preparation, the ascending thoracic aorta was excised and cut into 2–3 mm sections before mounting on wires for force measurements.

### Measurement of Bronchial Diameter Changes

Experiments using mouse lung slices were conducted as described (Sanderson, 2011). Eight-week-old Balb/C mice were sacrificed by deep isoflurane anesthesia followed by cervical dislocation. Trachea were isolated, cannulated with a blunt needle and the lungs filled with 3 ml of Hanks balanced salt solution (HBSS) with 2% low-temperature melt agarose at 37°C followed by 0.5 ml of air to clear the airways of agarose. The mouse thoracic cavity was exposed and the lungs chilled with an ice bath. After gelling of the agarose, the lungs were removed and 200 μm sections made with a Leica Vibratome VT1200. The sections were placed in DMEM in a tissue-culture incubator at 37°C for up to 8 h. Experiments measuring diameter changes were conducted using an inverted 10X phase contrast objective and oblique lighting in a constant perfusion (0.5 ml/min) bath of HBSS at 22°C solution. Contractions were evoked by perfusion with 0.5 μM carbachol in HBSS followed by washout to precarbachol size. The effects of UCL 1684 were determined by perfusion with 5 μM UCL 1684 in HBSS for 5 min, followed by combined carbachol plus UCL 1684 and compared to carbachol-induced diameter change alone.

### Tracheal Smooth Muscle Cell Calcium Imaging

Rat trachea were isolated as described above, except that a guillotine was used for sacrifice instead of cervical dislocation. The dorsal muscle layer was removed from the hyaline cartilage rings and minced into ~1-mm fragments in Ca^2+^-free HEPES-buffered Krebs solution (140 mM NaCl, 4.7 mM KCl, 1.13 mM MgCl_2_, 10 mM HEPES, 10 mM glucose, pH 7.3). The tracheal smooth muscle (TSM) fragments were digested with 2.5 U/ml papain (MP Biomedicals) with 1 mg/ml BSA fraction V and 1 mg/ml dithiothreitol at 37°C on a rocking platform (250 rotations/min) for 20 min. After washing once with the Ca^2+^-free Krebs solution the TSM fragments were digested with 12.5 U/ml of type VII collagenase (Sigma Chemical) for 10 min on a rocking platform at 37°C, washed and centrifuged (750 G for two min.) three times in Ca^2+^-free Krebs-BSA solution and gently triturated up to 5 min to disburse single tracheal myocytes. TSM cells were stored on ice in Ca^2+^-free Krebs-BSA solution and used the same day.

TSM cells were placed on a coverslip in a 1 ml perfusion chamber on an inverted microscope and incubated with 20 μM Cal-520 AM dye (AAT Bioquest, Sunnyvale, California, USA) in 0.2% Pluronic F-127 and 1 mM probenecid in HBSS at room temperature for 30 min. The chamber was fitted with a top adaptor and coverslip (Warner Instruments, RC37WC) to establish a closed perfusion chamber. The cells were imaged at 20 second intervals using 488 nm excitation on a Nikon swept-field confocal microscope using slit-scan mode, 2×2 Binning, and 100 millisecond frames.

Due to the loss of functional muscarinic receptors in primary bronchial smooth muscle cells in culture, calcium imaging was performed in human primary bronchial smooth muscle cells (ScienCell, passage 3) stably transfected with the human M3 muscarinic receptor. Human M3 receptor transfected clones were stably selected with G418, and individual clones were expanded to a 10 cm dish and frozen into aliquots, and analyzed after a maximum of two additional passages.

### Assay of Gq-Coupled Receptor Activation

**Figure 1:**
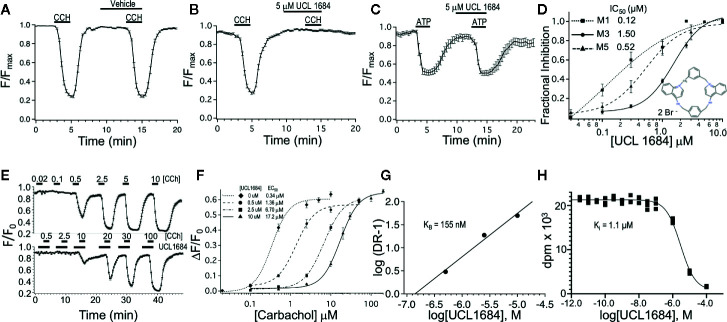
UCL 1684 is an antagonists for muscarinic acetylcholine receptors, but not purinergic receptors. **(A)** R-CEPIAer response to repeated 0.5 μM carbachol administration before and after vehicle (0.1% DMSO). R-CEPIAer fluorescence is normalized to maximal fluorescence (F/F_max_). Data is plotted for mean ± standard error of the mean. Fractional inhibition of second CCH response with vehicle was 0.0004 ± 0.018 of first response for N = 30 cells. **(B)** Same as in A except that 5 μM of UCL 1684 was applied preceding and during the second 0.5 μM carbachol administration. Fractional inhibition by UCL 1684 was 0.99 ± 0.011 for N = 30 cells. **(C)** R-CEPIAer response to repeated 1 μM ATP administration to CHO cells co-transfected with the human P2Y1 purinergic receptor. The second ATP administration included 5 μM UCL 1684. Fractional inhibition by UCL 1684 was 0.067 ± 0.06 for N = 20 cells. **(D)** R-CEPIAer dose-response to 0.5 μM CCH with increasing doses of UCL 1684. Data is plotted as mean fractional inhibition of 0.5 μM CCH response as a function of UCL 1684 concentrations. Inset is the molecular structure of UCL 1684 (from (Campos Rosa et al., 1996). Fitting of the inhibition curves to a Hill equation indicated IC_50_ and slope values of the following: M1 receptor 0.12 ± 0.18 μM, slope 0.67 ± 0.5, N=55–61; M3 receptor 1.5 ± 0.14 μM, slope 1.8 ± 0.29, N=28–30; M5 receptor 0.52 ± 0.05 μM, slope 1.4 ± 0.17, N=58. **(E)** R-CEPIAer response in M3 mACHR expressing cells to increasing carbachol administration in the absence (above) and presence (below) of 10 μM UCL 1684. Data is plotted as mean ± s.e.m. for N =19 for control, and N=21 for 10 μM UCL 1684. **(F)** ΔF/F_0_ R-CEPIAer response M3 mACHR expressing cells as in (E), were plotted for increasing carbachol concentrations alone (diamond, EC_50_ 0.34 ± 0.06, slope 2.0 ± 0.65, N=19), or carbachol with UCL1684: 0.5 μM (circle, EC_50_ 1.36 ± 0.26, slope 1.9 ± 0.45, N=20), 2.5 μM (square, EC_50_ 6.70 ± 0.47, slope 1.8 ± 0.18, N=20) and 10 μM (triangle, EC_50_ 17.2 ± 0.90, slope 1.9 ± 0.20, N=21). **(G)** Schild analysis of M3 receptor inhibition by UCL1684. EC_50_ values from F were used to plot log of Dose Ratio -1, (DR, carbachol EC_50_ value in the presence/EC_50_ absence of UCL1684) as a function of log of UCL1684 concentration. Linear regression fitting yielded a slope of 0.94, y-intercept of 6.4, and a theoretical X-intercept yielding a pKi of 6.8 (*K*_i_ of 155 nM). **(H)** A representative binding experiment using M3 mACHR-expressing CHO cell membranes (25 ug) incubated with 0.9 nM [^3^H]-NMS and indicated concentrations of UCL1684 (as described in Methods). The experiment shown indicated a pKi of 5.95 (Ki 1.1 μM). The average pKi was 6.04 ± 0.04 (909 nM, Mean ± SEM, n=3 separate experiments).

The binding of antagonists to Gq-coupled receptors was determined by measuring IP3-mediated calcium depletion from the endoplasmic reticulum (ER). This was accomplished using CHO-K1 cells stably transfected with human M3 muscarinic acetylcholine receptor (CEM3000000, cDNA Resource Center, Bloomsburg University) and R-CEPIAer, a low affinity ER-targeted calcium indicator fluorescent protein (Suzuki et al., 2014). R-CEPIAer reporting of ER calcium concentration was assessed using 564 nm excitation light (>590 long-pass emission) at 20 second intervals on a Nikon swept-field confocal microscope using slit-scan mode, 2x2 Binning, and 100 millisecond frames. F/F_max_ was fluorescence intensity normalized to maximal fluorescence intensity during the experiment. The response of cells to Gq-coupled receptor agonist were measured as the change in F/F_max_ (ΔF/F_max_) from preagonist value to fluorescence minimum during agonist application (that occurs by IP3-mediated ER calcium depletion). This generally resulted in a ΔF/F_max_ of ~ 0.8–0.7 in most experiments using 0.5 μM carbachol (i.e. **Figure 1A**). Experiments were generally paired to compare a first control response to carbachol (or other agonist), to a second carbachol response with antagonist (i.e. **Figure 1A**). Perfusion of each agonist application was for 2 min, while the antagonist overlapped and preceded the second agonist application by 3 min. The second ΔF/F_max_ response with antagonist was divided by the first ΔF/F_max_ without antagonist to obtain a fractional inhibition value, with no inhibition having a theoretical value of 0 (i.e. response to 0.1% DMSO was 0.0004, **Figure 1A**) and complete inhibition having a value of 1 (i.e. 5 μM UCL 1684 was 0.99, **Figure 1B**). IC_50_ was calculated by assaying fractional inhibition using a dose-response of antagonist concentrations (i.e. **Figure 1D**). For assays of other receptors such as the M1 and M5 muscarinic receptors, H1 histamine and P2Y1 purinergic receptors, these receptors were transiently co-transfected with the pCMV R-CEPIAer plasmid (a gift from Masamitsu Iino (Addgene plasmid # 58216; http://n2t.net/addgene:58216; RRID : Addgene_58216)) into CHO cells using polyethylenimine [according to previous methods (Longo et al., 2013)]. IC_50_ and EC_50_ values were estimated by fitting the dose-response relationship to a Hill equation.

To obtain a pK_B_ using a Schild analysis (Arunlakshana and Schild, 1959), the acetylcholine EC_50_ was measured in the absence of UCL1684 and EC_50′_ measured in the presence of increasing concentrations of UCL1684. These values were used to calculate a dose ratio (DR = EC_50_′/EC_50_) for each UCL1684 concentration. The log(DR − 1) was plotted vs. log UCL1684 concentrations and fit to a linear equation where the theoretical X-intercept was used to estimate a pK_B_.

### Radioligand Binding

Competition binding assays were done with [^3^H]-N-methyl-scopolamine ([3H]-NMS, PerkinElmer) essentially as described (Steinfeld et al., 2010) with minor modifications. Membrane homogenates were prepared in HEPES assay buffer (20 mM HEPES, 100 mM NaCl, and 10 mM MgCl, pH 7.4) by trituration on ice through a 26-gauge needle followed by centrifugation at 39,000g in Sorval RC-6+ centrifuge. Membranes were resuspended in assay buffer and protein concentration determined by the method of Bradford using bovine serum albumin as a standard (Bradford, 1976). Membranes (25 µg) were incubated along with 0.9 nM ^3^H- NMS without or with UCL1684 concentrations (10^-11^M to 10^-4^M in triplicate) for 120 min at 30°C in 96 multiwell GF/C filter plates (MilliporeSigma) coated with 0.3% polyethyleneimine (Sigma). The assay was terminated by rapid filtration using a MilliporeSigma vacuum manifold followed by three washes with ice-cold wash buffer (20mM HEPES, pH 7.4). Filters were removed and placed in 7 ml scintillation vials containing 5 ml scintillation fluid (National Diagnostics) and radioactivity counted using a Beckman LS-6500 ß-counter. Nonspecific binding was defined by 100 µM atropine. The Ki value for UCL1684 was determined for each of three separate binding assays using the one-site fit Ki equation with Prism software (Graphpad, version 8) where the Kd for [^3^H]-NMS was constrained to 0.56 nM (Steinfeld et al., 2010).

### Data Analysis

Igor 5 (Igor, WaveMetrics Inc.), KaleidaGraph 4.1.1 (Synergy Software), and Excel 2007 (Microsoft Corp.) were used for statistical analyses. Significance was determined with paired or unpaired t-test. The effects were deemed significant when a P < 0.05 was obtained. The results are expressed as the means ± standard error of means where applicable.

## Results

### Inhibition of M3 Muscarinic Receptors

CHO cells were stably transfected with human M3 muscarinic acetylcholine receptors and R-CEPIAer reporter of ER calcium. M3 muscarinic receptor activation causes Gq signaling that in turn leads to IP3-mediated calcium depletion from the ER (Edelman et al., 1994), which can be assayed using the R-CEPIAer fluorescent reporter (Suzuki et al., 2014). In paired experiments, a reproducible depletion of ER calcium was seen upon repeated application of muscarinic receptor agonist 0.5 μM carbachol (CCH) without and with vehicle (0.1% DMSO, **Figure 1A**). However, UCL 1684 (5 μM) preceding and during the second application of 0.5 μM CCH completely blocked the muscarinic response (**Figure 1B**, fractional inhibition 0.99 ± 0.011). The antagonist effect was specific to the M3 muscarinic receptor, and not downstream Gq signaling since UCL 1684 did not block the effect of other Gq-coupled receptors such as purinergic receptor P2Y1 (**Figure 1C**, fractional inhibition 0.067 ± 0.06) or histamine receptor H1 (data not shown, fractional inhibition -0.008 ± 0.04). The dose-response relationship between UCL 1684 and CCH-evoked calcium release was investigated also using paired experiments (as in **Figure 1B**) and varying the UCL 1684 concentration. A half maximal concentration of 1.5 μM UCL 1684 blocked the response of M3 muscarinic receptors to 0.5 μM CCH (**Figure 1D**). UCL 1684 also blocked activation of the related M1 and M5 muscarinic receptors with IC_50_’s of 0.12 μM and 0.52 μM, respectively (**Figure 1D**).

The nature of the antagonism of UCL 1684 on muscarinic receptors was investigated by assaying the effect of UCL 1684 on the CCH dose-response. CCH-dose responses comparing control (top, **Figure 1E**) to 10 μM UCL 1684 treatment (bottom, **Figure 1E**) reveal a reduced sensitivity of receptors to CCH with UCL 1684. Summary data comparing several UCL1684 concentrations demonstrate a rightward shift of the CCH dose-response with increased concentrations of UCL1684, without a change in the maximal response (**Figure 1F**). This suggests that UCL 1684 acts as a competitive antagonist for CCH binding to muscarinic receptors (Thorsteinn, 2015). The data was evaluated using a Schild analysis (**Figure 1G**) which indicated a linear relationship (slope 0.94) consistent with competitive antagonism and an estimated equilibrium binding constant (K_B_) of 155 nM. The affinity of UCL1684 for M3 receptors expressed in CHO cells was also determined in competition binding assays using [^3^H]-N-methyl-scopolamine (example experiment in **Figure 1H**). The pKi was 6.04 ± 0.04 (909 nM, Mean ± SEM, n=3).

**Figure 2:**
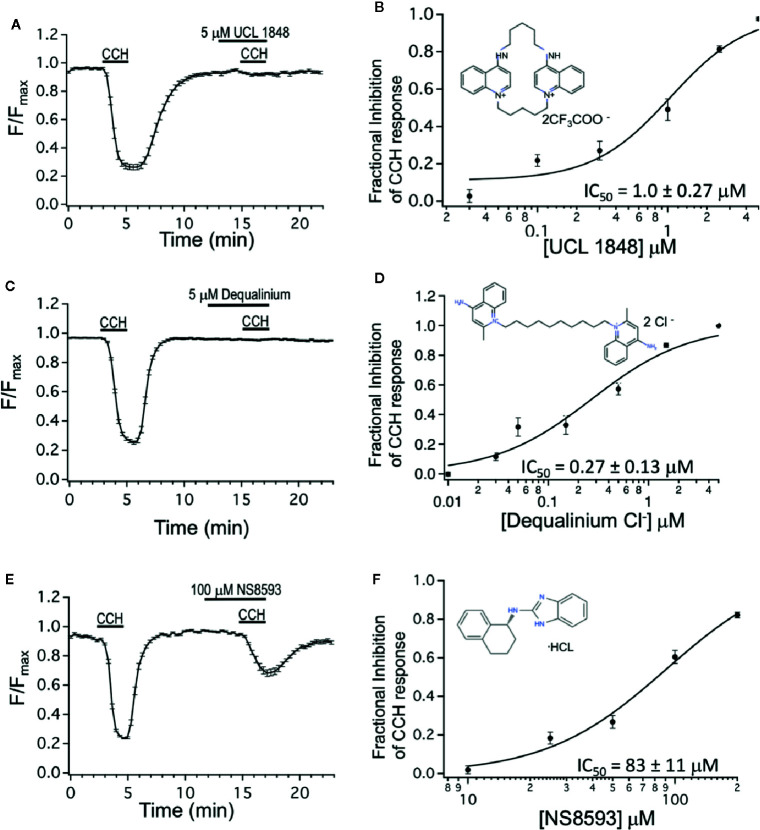
Dequalinium compounds are M3 muscarinic receptor blockers. **(A)** R-CEPIAer response to repeated 0.5 μM carbachol administration with 5 μM of UCL 1848 that was applied 3 min preceding and during the second 0.5 μM carbachol administration. Fractional inhibition to UCL 1848 was 0.98 ± 0.01 for N = 32 cells. **(B)** Mean fractional inhibition of 0.5 μM CCH response as a function of UCL 1848 concentration (N = 20–32 cells per dose). Inset is the molecular structure of UCL 1848 (Chen, Galanakis et al., 2000). Fitting of data to a Hill equation revealed an IC_50_ of 1.0 ± 0.27 μM, slope 1.47 ± 0.46. **(C)** R-CEPIAer response to repeated 0.5 μM carbachol administration with 5 μM of dequalinium chloride that was applied 3 min preceding and during the second 0.5 μM carbachol administration. Fractional inhibition to dequalinium was 0.99 ± 0.003 for N = 42 cells. **(D)** Mean fractional inhibition of 0.5 μM CCH in response as a function of dequalinium chloride concentration (n=15–42 cells per dose). Inset is the molecular structure of dequalinium chloride (Chen et al., 2000). Fitting of data to a Hill equation revealed an IC_50_ of 0.27 ± 0.13 μM, slope 0.90 ± 0.29. **(E)** R-CEPIAer response to repeated 0.5 μM carbachol administration with 100 μM of NS8593 applied 3 min preceding and during the second 0.5 μM CCH administration. Fractional inhibition to NS8593 was 0.60 ± 0.03 for N = 30 cells. **(F)** Mean fractional inhibition of 0.5 μM CCH response as a function of NS8593 concentration (N=30–34 cells per dose). Inset is the molecular structure of NS8593 (Sorensen et al., 2008). Fitting of data to a Hill equation revealed an IC_50_ of 83 ± 11 μM, slope 1.7 ± 0.35. All dose-response experiments were conducted with 0.5 μM carbachol concentration.

We also tested the UCL 1684-related bis-quinolinium cyclophane, UCL 1848 (Campos Rosa et al., 1996). **Figure 2A** shows that 5 μM UCL 1848 fully prevented the CCH-evoked calcium depletion (**Figure 2A**), and the dose-response experiment revealed that UCL 1848 blocked the muscarinic response with an IC_50_ of 1.0 μM (**Figure 2B**). As well, dequalinium chloride effectively blocked M3 receptor activation (**Figure 2C**) with an IC_50_ of 0.27 μM (**Figure 2D**). As a control, we also investigated an unrelated SK channel antagonist NS8593, which antagonizes SK channel at submicromolar concentrations through effects on calcium sensing rather than pore blocking as UCL 1684 (Strobaek et al., 2006; Sorensen et al., 2008). As expected, NS8593 dose-response curve indicates that effects of micromolar concentration (10 μM) was undetectable (**Figure 2F**), but we nevertheless could see partial antagonist effect at very high concentrations (100 μM, **Figure 2E**) with an estimated IC_50_ of 83 μM (**Figure 2F**).

### Inhibition of Smooth Muscle Contraction

**Figure 3:**
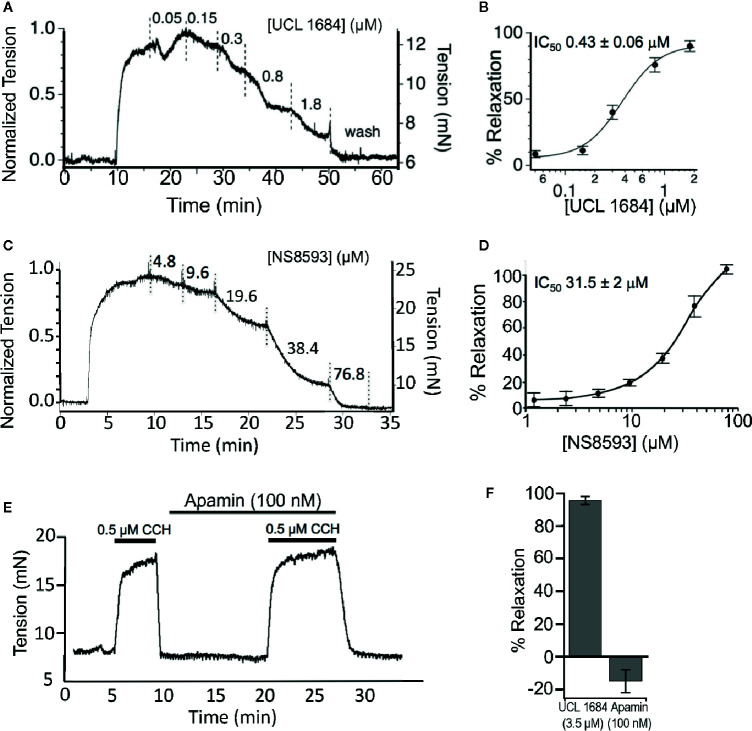
UCL 1684 relaxes cholinergic evoked airway smooth muscle contractions. **(A)** Example isometric tension measurement of mouse trachea. The trachea was precontracted with 0.5 μM carbachol and cumulative increasing concentrations of UCL 1684 was applied to the bath at approximately 5-min intervals (or until tensions approached steady state levels). **(B)** Average response from N = 5 tracheas. The IC_50_ was estimated to be 0.43 ± 0.06 μM, slope 1.7 ± 0.32 using a Hill function to the average response. **(C)** An example cumulative dose-relaxation response to increasing concentrations of NS8593. **(D)** Average response from N = 4–8 tracheas (depending on dose). The IC_50_ was estimated to be 31.5 ± 2.0 μM, slope 1.3 ± 0.22 using a Hill function to the average response. **(E)** Example contraction in response to 0.5 μM carbachol, without and with wash-in of 100 nM of Apamin. **(F)** Summary comparisons of relaxation to 0.5 μM carbachol using single doses of 3.5 μM UCL 1684 (95.4 ± 2.4, N=6), and 100 nM Apamin (−15 ± 7, N=4).

Given that M3 muscarinic acetylcholine receptors play a key role in regulating airway smooth muscle (ASM) contractility, we investigated UCL 1684 physiological effects on muscle contractility. We used isolated mouse tracheal rings and measured isometric force in an organ bath to determine if drugs modulate ASM contractions. Tracheas were precontracted with a half-maximal concentration (0.5 μM) of CCH, and relaxation was assayed using increasing cumulative concentrations of UCL 1684 (**Figure 3A**). We found that UCL 1684 caused relaxation, with half-maximal concentration of 0.43 μM (**Figure 3B**), and near complete relaxation with 1.8 μM. Consistent with the pharmacology data (**Figure 2C**), NS8593 caused relaxation at much higher concentrations (**Figure 3C**). On average, NS8593 had a half maximal concentration of 31 μM and caused complete relaxation at the highest concentration of 76.8 μM (**Figure 3D**). Consistent with the drug action that is independent of SK channels, peptide blocker of SK channels, Apamin (100 nM), did not relax ASM contractions (**Figure 3E**), and even increased contractions slightly (summarized in **Figure 3F**).

**Figure 4:**
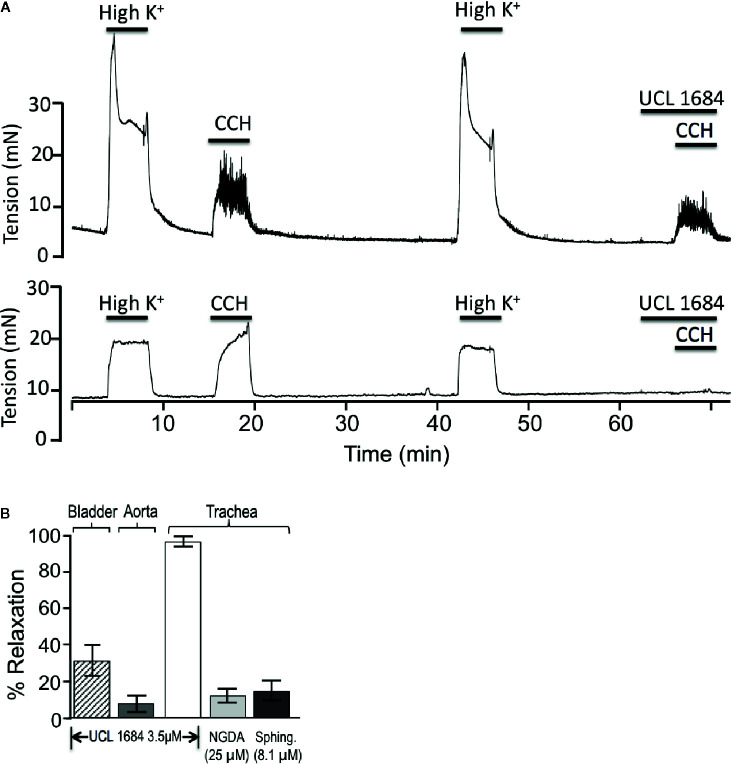
UCL 1684 potently relaxes airway smooth muscle, but has weak effects on bladder and vascular muscle. **(A)** Sample tracings of bladder (above) and trachea (below) response to 3.5 μM UCL 1684. Shown are repetitive contractile responses to high potassium stimulation (67 mM K^+^ PSS solution) and 0.5 μM carbachol. The second carbachol response was preceded by addition of UCL 1684, which had moderate effects on bladder, but completely prevented trachea contraction response. **(B)** Summary 3.5 μM UCL 1684 relaxation response (as above) of bladder (diagonal hatch, 31.4 ± 8.4, N=6), aorta precontracted to 300 nM phenylephrine (7.8 ± 4.3, N=3), and trachea (white, 95.4 ± 2.4, N=6). Trachea was also tested for relaxation response to TrpM channel blockers nordihydroguaiaretic acid (NGDA, light gray, 12.4 ± 8.5, N=8), and d-erthyro-sphingosine (Sphing., dark gray, 14.9 ± 6.0, N=8).

We investigated if UCL 1684 relaxation of smooth muscle also acted on other known cholinergic responsive (bladder) or cholinergic non-responsive (vascular) smooth muscle (Herrera and Nelson, 2002; Thorneloe et al., 2008; Li et al., 2017). Pretreatment with UCL 1684 partially inhibited relaxation of bladder contraction (**Figure 4A**, top panel, summarized in 4B), and caused little inhibition of phenylephrine-evoked contraction of vascular (aortic) rings (summarized in **Figure 4B**).

Others have reported that both UCL 1684 and NS8593 also inhibit TrpM3 and TrpM7 channels (Chubanov et al., 2012). We therefore investigated if using saturating concentrations of TrpM channel antagonist would have similar effects as UCL 1684. We applied inhibitors of TrpM3/7 channels, including 25 μM nordihydroguaiaretic acid (NGDA) (Chen et al., 2010), and 8.1 μM Sphingosine (Qin et al., 2013), and observed small effects on airway contraction (**Figure 4B**).

**Figure 5:**
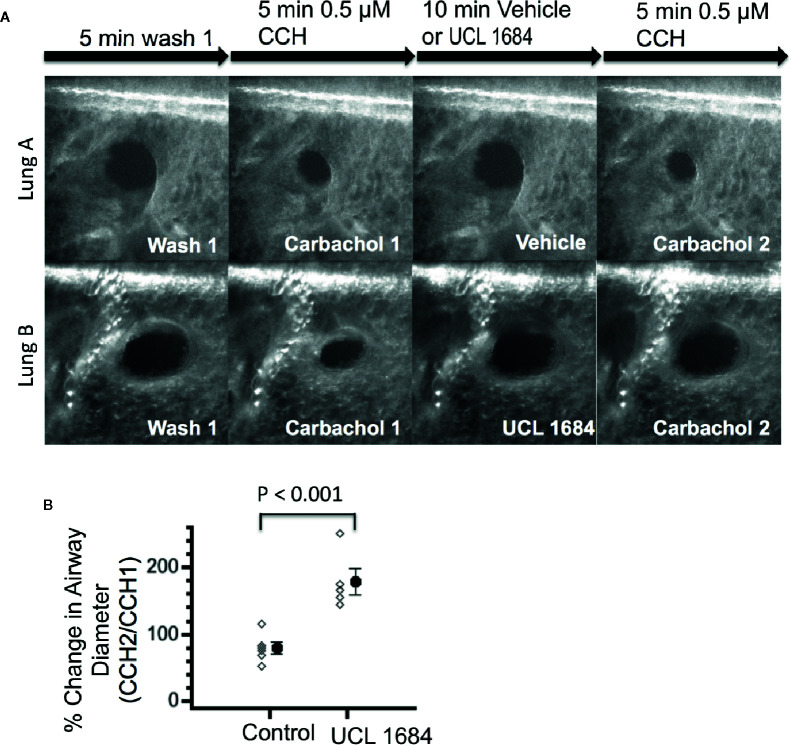
UCL 1684 causes relaxation of mouse lung lower airways. **(A)** Example of reproducible bronchoconstriction in response to repeated exposure of 0.5 μM carbachol (top row). Identical experiments with additional superfusion of 3.5 μM UCL 1684 preceding second carbachol challenge (bottom row) indicates that UCL 1684 prevents bronchoconstriction. **(B)** Summary data of bronchial diameter during second carbachol challenge normalized to first challenge (in percentage) was 79.0 ± 8.5, N=6 for control, and 178.3 ± 19.0, N=5 for UCL 1684 treated. P < 0.001 in Student’s t-test.

To investigate if UCL 1684 could function on lower airways smooth muscle, we measured bronchial diameter changes in precision cut mouse lung slices and in response to cholinergic activation. **Figure 5A** shows a control experiment (upper row) where airway diameter was compared with repeated treatment of 0.5 μM CCH. After washout of a first CCH administration, vehicle pretreatment and the second CCH administration shows a similar diameter, which was 79.0% of the first CCH response (summarized in **Figure 5B**). Application of UCL 1684 preceding the second CCH administration inhibited bronchial constriction, as the diameter was on average 178.3 larger than CCH treatment alone (**Figure 5A** lower row, summarized in **Figure 5B**).

### Inhibition of Smooth Muscle Calcium Release

**Figure 6:**
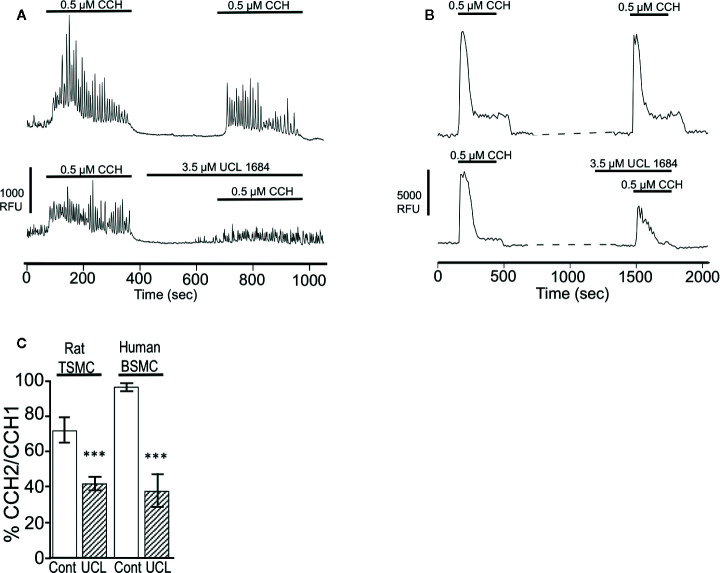
UCL 1684 causes a reduction of cholinergic-evoked calcium release. **(A)** Representative cytoplasmic calcium response of acutely isolated rat tracheal smooth muscle cells to repetitive 0.5 μM carbachol application without (upper panel) and with 3.5 μM UCL 1684 preceding second carbachol challenge (bottom panel). **(B)** Representative cytoplasmic calcium response of human bronchial smooth muscle cells to repetitive 0.5 μM carbachol application without (upper panel) and with 3.5 μM UCL 1684 preceding second carbachol challenge (bottom panel). **(C)** Summary data from A and B. Data is the average percent change of calcium response (integral) to repeated carbachol treatment without (white column) and with UCL 1684 (hashed column) preceding the second challenge. Measurements were from tracheal smooth muscle cells (TSMC, as in A) and human bronchial smooth muscle cells (BSMC, as in B). Rat TSMC control was 0.72 ± 0.07, N=27, UCL 1684 treated was 0.42 ± 0.04, N=40, P < 0.001. Human BSMC control was 0.97 ± 0.03, N=25, UCL 1684 treated was 0.38 ± 0.10, N=16, P < 0.0001. Comparisons were analyzed using a paired Student’s t-test.

M3 muscarinic receptors mediate smooth muscle contraction through IP3-mediated calcium release (Fryer and Jacoby, 1998). The effect of UCL 1684 on calcium release was assessed in rat primary tracheal smooth muscle cells using the calcium-sensitive dye AM-Ca520 to measure cytosolic calcium. We found that individual cells had variable responses to cholinergic activation, with some cells showing extensive calcium oscillations while others showing few. We therefore compared CCH-evoked calcium release before and after treatment within the same cell. Overall, UCL 1684 reduced CCH-evoked cytosolic calcium to 42% of control (**Figure 6A**, lower panel and summarized in **Figure 6C**), which was significantly larger effect than then the 72% of vehicle treatment alone (**Figure 6A**, upper panel, summarized in **Figure 6C**). We also conducted similar experiments in early passage human bronchial smooth muscle cells (BSMC, **Figure 6B**) which showed a more reproducible response to repeated CCH administration. Vehicle treatment caused a second response that was 97% of control (**Figure 6B**, upper panel). However, UCL 1684 significantly reduced CCH-evoked cytosolic calcium to 38% of control (**Figure 6B** lower panel, summarized in **Figure 6C**).

## Discussion

Our key finding is that dequalinium compounds are antagonists of M3 muscarinic acetylcholine receptors at low micromolar to nanomolar concentrations (**Figure 1**). Schild analysis of the calcium release data indicates that UCL1684 is a competitive antagonist for cholinergic binding to M3 receptors with a K_B_ of 155 nM. However, the affinity (Ki) determined from competition binding experiments indicate Ki of was much lower (900 nM). This difference in affinity measurements may simply be due to the fact that binding equilibrium was not achieved in the calcium release assay resulting in overestimation of UCL 1684 affinity. In contrast, UCL 1684 and UCL 1848 have higher affinity to SK potassium channels with IC_50_ of 3 nM and 2 nM, respectively, as determined by effects on SK-dependent fast-afterhyperpolarizing currents (Campos Rosa et al., 1996; Chen et al., 2000; Blank et al., 2004). Radioligand competition assays (using ^125^I-Apamin) similarly indicate high affinity binding of UCL1684 with Ki of 1 nM for SK2 and 7.7 nM for SK3 channels (Benton D.C. et al., 2013). Despite high affinity to SK channels, micromolar concentrations of UCL 1684 are sometimes used to overcome diffusion issues through tissues or brain slices (Kroigaard et al., 2012; Benton M. D. et al., 2013; Chien and Su, 2015; Mader et al., 2016) or perhaps when administering to whole animals (Diness et al., 2011). The consequence is that these drugs may also inadvertently block muscarinic responses. Indeed, we found M1 receptors were particularly sensitive to UCL 1684 with an IC_50_ of 0.12 μM (**Figure 1D**).

Poorly absorbed quaternary ammonium-inhaled muscarinic antagonists such as ipatroprium (short acting), and tiotropium (long acting) have long been used as inhaled bronchodilators for chronic obstructive pulmonary disease (Ariel et al., 2018; Yamada and Ichinose, 2018), and more recently as an adjunct therapy for asthma (Mansfield and Bernstein, 2019). Given that UCL 1684 is also a muscarinic receptor antagonist, we examined its effects on airway contractions. We indeed found that UCL 1684 acts at submicromolar concentrations to reduce cholinergic-evoked calcium release, and thereby relax airway smooth muscle. UCL 1684 was found to have sustained effects and required at least an hour of washout to restore tracheal responses to cholinergic agonists (data not shown). Further, one might hypothesize that the reduced calcium release may have the added benefit of preventing asthma induced airway remodeling that is dependent on calcium (Sweeney et al., 2002; Kellner et al., 2008; Mahn et al., 2010).

Dequalinium salts have been used extensively for their broad bactericidal and fungicidal activity (Tischer et al., 2012). These compounds are used as topical antimicrobials (Neodequin), for mouth infections (Dequadin) and also for vaginal bacterial infections (Fluomizin). Dequalinium chloride has relatively low toxicity *via* oral administration (LD_50_ in mouse of 300 mg/kg), but LD_50_ 18.3 mg/kg in mouse with intraperitoneal administration (Gamboa-Vujicic et al., 1993). Interestingly, the sore throat and mouth side-effects seen for Dequadin could be due to its antimuscarinic activity that is a common side effect due to reduced oral secretions. It is possible that dequalinium chloride, if accessible to target organs, might be a useful alternative to other antimuscarinics such as treatment for bronchoconstriction, pupil dilation, motion sickness, bradycardia, and overactive bladder (Eglen et al., 2001; Ellsworth, 2012; Madersbacher et al., 2013; Bostock and McDonald, 2016; Matera and Cazzola, 2017).

## Data Availability Statement

The raw data supporting the conclusions of this article will be made available by the authors, without undue reservation.

## Ethics Statement

The animal study was reviewed and approved by UT Health San Antonio Institutional Care and Use Committee.

## Author Contributions

RB, PD, KB, and EB designed the experiments. RB wrote and finalized the manuscript. VB, DW, BW, IS, HS, and PC conducted experiments and data analysis. All authors read and approved the final manuscript.

## Funding

This work was supported by NIH grants AI113724, DA038645, DA048214; NSF grant 1456862, and the Welch Foundation grant AQ-1980-20190330.

## Conflict of Interest

The authors declare that the research was conducted in the absence of any commercial or financial relationships that could be construed as a potential conflict of interest.

## Abbreviations

BSA, bovine serum albumin; Ca^2+^, calcium; CCH, carbachol; IC_50_, the concentration necessary to reduce a response by half; EC_50_, the concentration that gives half-maximal response; HBSS, Hanks balanced salt solution; PSS, physiological saline solution; TSM, tracheal smooth muscle; SK, small-conductance, calcium-activated potassium channel; UCL 1684: 6,12,19,20,25,26-Hexahydro-5,27:13,18:21,24-trietheno-11,7-metheno-7*H*-dibenzo [*b*,*n*] [1,5,12,16]tetraazacyclotricosine-5,13-diium dibromide; UCL 1848: 8,14-Diaza-1,7(1,4)-diquinolinacyclotetradecaphane trifluoroacetate salt; NS8593: *N*-[(1*R*)-1,2,3,4-Tetrahydro-1-naphthalenyl]-1*H*-benzimidazol-2-amine hydrochloride, dequalinium chloride: 1-[10-(4-imino-2-methyl-1,4-dihydroquinolin-1-yl)decyl]-2-methyl-1,4-dihydroquinolin-4-imine dihydrochloride.
